# The interfaces of lanthanum oxide-based subnanometer EOT gate dielectrics

**DOI:** 10.1186/1556-276X-9-472

**Published:** 2014-09-05

**Authors:** Hei Wong, Jian Zhou, Jieqiong Zhang, Hao Jin, Kuniyuki Kakushima, Hiroshi Iwai

**Affiliations:** 1 Department of Electronic Engineering and Information Sciences, Zhejiang University, Hangzhou, China; 2 Department of Electronic Engineering, City University of Hong Kong, Tat Chee Avenue, Kowloon, Hong Kong; 3 Frontier Research Center, Tokyo Institute of Technology, Yokohama 226-8502, Japan

**Keywords:** High-*k*, Lanthanum oxide, Si/high-*k* interface, Metal gate/high-*k* interface

## Abstract

When pushing the gate dielectric thickness of metal-oxide-semiconductor (MOS) devices down to the subnanometer scale, the most challenging issue is the interface. The interfacial transition layers between the high-*k* dielectric/Si and between the high-*k* dielectric/gate metal become the critical constraints for the smallest achievable film thickness. This work presents a detailed study on the interface bonding structures of the tungsten/lanthanum oxide/silicon (W/La_2_O_3_/Si) MOS structure. We found that both W/La_2_O_3_ and La_2_O_3_/Si are thermally unstable. Thermal annealing can lead to W oxidation and the forming of a complex oxide layer at the W/La_2_O_3_ interface. For the La_2_O_3_/Si interface, thermal annealing leads to a thick low-*k* silicate layer. These interface layers do not only cause significant device performance degradation, but also impose a limit on the thinnest equivalent oxide thickness (EOT) to be achievable which may be well above the requirements of our future technology nodes.

## Background

In the deca-nanometer complementary metal-oxide-semiconductor (CMOS) devices, the thickness of the gate dielectric film should be scaled down to the subnanometer equivalent oxide thickness (EOT) range in order to have proper control of the channel current under a reasonable gate bias [1-3]. This ultimate dielectric thickness requirement imposes a number of challenges on both the fabrication process and the device characteristic optimization. Interface properties and their thermal instabilities turn out to be the major challenging issues. Transition metal (TM)- or rare earth metal (RE)-based high-*k* dielectrics are extrinsic materials to the substrate silicon; they can react with silicon at some elevated temperatures [4-8], and the chemical reactions at the high-*k*/silicon interface cause most of the performance degradation issues. Conventional MOS layout for large-scale integration is in the planar structure, and the channel mobility of the transistors is predominately governed by the dielectric/silicon interface. Improvement of the SiO_2_/Si interface property had been one of the major concerns since the invention of the MOS transistor regardless of the fact that the SiO_2_/Si interface is already almost perfect as it is grown thermally in a self-organizing way from the intrinsic material [9-11], whereas the quality of the high-*k* metal/Si interface was found to be much poor. It was found that there exists a relative low-*k* transition layer between the TM/RE metal oxide and substrate silicon [12,13]. This low-*k* layer may be of several angstroms to a nanometer thick and may become the major portion of the subnanometer EOT dielectric film. This transition layer, which cannot be scaled down for thinner high-*k* films, has become the major challenging issue for the subnanometer EOT thin film [1,2]. The metal gate/high-*k* interface where a low-*k* transition layer may exist will also affect the resulting EOT; unfortunately, this issue was seldom studied. In this work, we took the tungsten/lanthanum oxide/Si (W/La_2_O_3_/Si) structure as an example to have a detailed study on the bonding structures together with thermal annealing effects on the W/La_2_O_3_ interface and La_2_O_3_/Si interface by employing combined angle-resolved X-ray photoelectron spectroscopy (ARXPS) and film thinning with *in situ* sputtering using an XPS source.

## Methods

The tungsten/La_2_O_3_ gate stack was deposited on the n-type Si (100). A La_2_O_3_ film of about 5 nm thick was prepared by electron beam evaporation in an ultra-high vacuum chamber with a pressure of about 10^−7^ Pa. A tungsten gate electrode of about 3 nm thick was then deposited *in situ* using magnetron sputtering to avoid any moisture absorption and contamination. Some samples were further thermally annealed at 600°C for 30 min in a rapid thermal annealing furnace. The chemical compositions as well as the bonding structures of the as-prepared W/La_2_O_3_/Si stack at different depths were investigated in detail by using a Physical Electronics PHI 5802 spectrometer (Physical Electronics, Inc., Chanhassen, MN, USA) with monochromatic Al Kα radiation with an energy of 1,486.6 eV. To study the bonding structure on both W/La_2_O_3_ and La_2_O_3_/Si interfaces, both depth profiling by argon sputtering and angle-resolved techniques were used.

## Results and discussion

### High-*k*/metal gate interface

**Figure 1:**
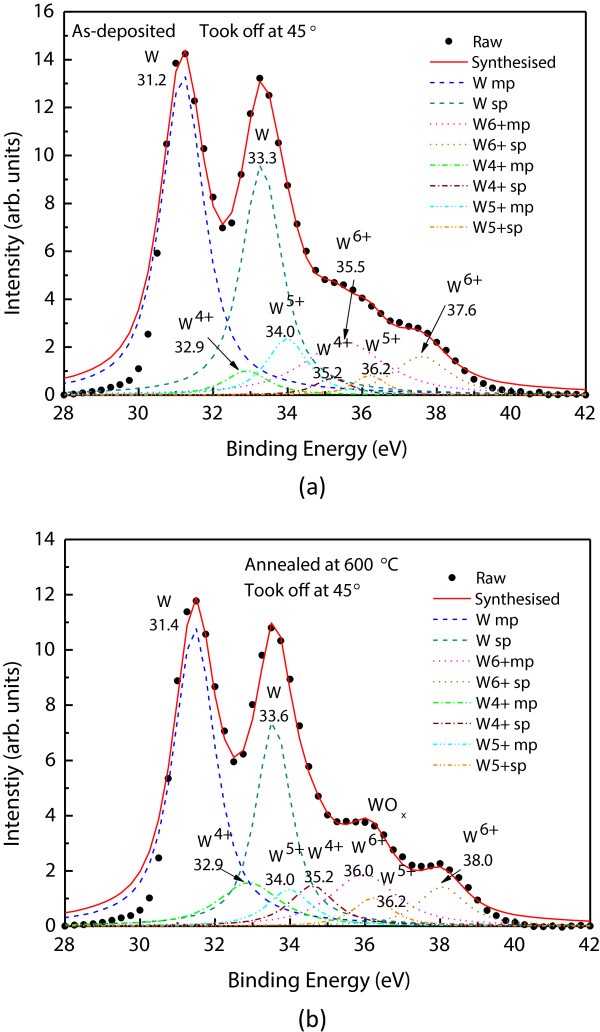
**W 4f XPS spectra with Gaussian decomposition.** This figure shows various oxidized states of tungsten near the W/La_2_O_3_ interface. **(a)** As-deposited film. **(b)** Sample with thermal annealing at 600°C for 30 min. A stronger WO_*x*_ peak was observed.

**Figure 2:**
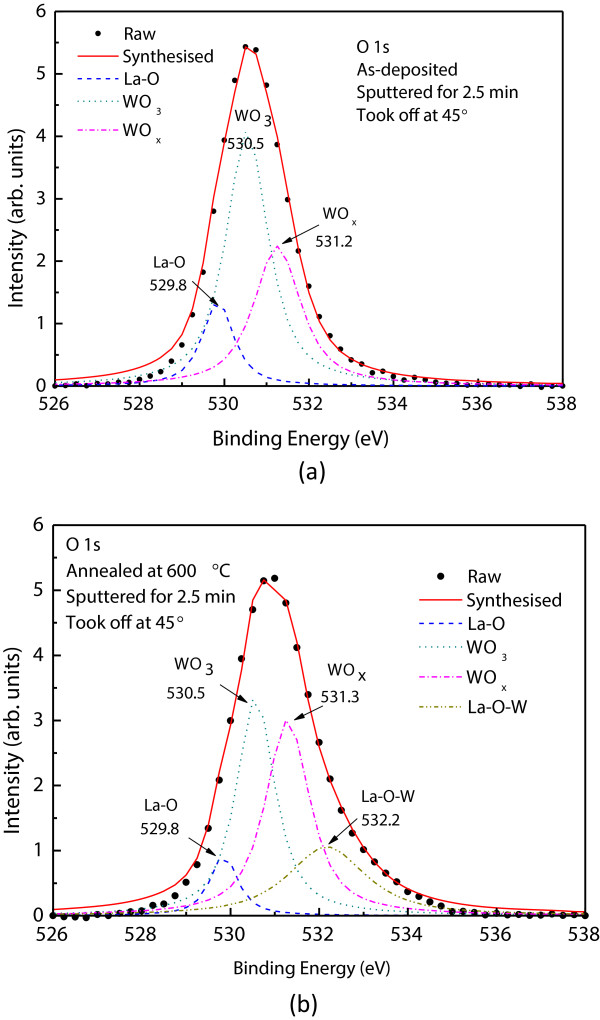
**O 1s spectra taken near the W/La**_**2**_**O**_**3 **_**interface. (a)** Three oxidation states, corresponding to WO_3_, WO_*x*_, and La-O, were found for the as-deposited film. **(b)** After thermal annealing, an additional peak, attributing to La-O-W bonding, was found at an energy of 532.2 eV.

The high-*k*/metal gate interfacial layer can be either an insulating layer or a conductive layer. For the conventional poly-Si gate, a thick insulating silicate layer can be formed. For the La_2_O_3_/Al stack, the interfacial layer is aluminum oxide or lanthanum aluminates. These interface layers generally have much smaller *k* values (<15) than the desired high-*k* gate dielectric. The thickness of this transition layer may range from 0.3 to over 1 nm depending on the material and the post high-*k* deposition temperature. With this low-*k* transition layer, subnanometer EOT is hard to be achieved. It will be good if the transition layer between metal/high-*k*, e.g., W/La_2_O_3_ stack, is conductive. By using angle-resolved XPS with take-off angle varying from 0° to 90° together with argon sputtering for film thinning, bonding details along the depth direction were obtained in this work. Oxidized tungsten phases were found both on the surface and at the W/La_2_O_3_ interface. Figure 1 depicts the W 4f XPS spectra taken from a W/La_2_O_3_ stack with a take-off angle of 45°. The elemental W has a doublet with energies at 31.2 and 33.3 eV. By employing Gaussian decomposition technique, several oxidized states were observed for both as-deposited and thermally annealed samples. These results indicate that there exist WO_*x*_ phases in the transition layer. The W atoms in WO_*x*_ form are in d^2^ configuration, and that makes the WO_*x*_ conductive. Thermal annealing at 600°C can enhance the W oxidation at the W/La_2_O_3_ interface significantly (see Figure 1b). These observations were further confirmed with the O 1s XPS spectra. Figure 2 shows the O 1s XPS for both as-deposited and 600°C annealed samples. Gaussian decomposition of the O 1s peak indicates that the oxygen in the as-deposited film has three main bonding states with energies of 528.9, 530.5, and 531.2 eV corresponding respectively to La-O, WO_3_, and WO_*x*_ bonding. After thermal annealing at 600°C for 30 min, the WO_*x*_ phase was significantly enhanced. The oxygen for interface W oxidation should come from the La_2_O_3_ film. It was proposed that the oxygen in W may diffuse into the La_2_O_3_ film to fill up the oxygen vacancies there [14]. Oxygen vacancies are the major defect centers in La_2_O_3_ which result in several instability issues and enhance the gate leakage current [15-17]. The present result indicates that a reverse process may have been taken place in the present samples. That means a high-temperature process may lead to the out-diffusion of oxygen to the W/La_2_O_3_ interface, and that increases oxygen vacancies in the La_2_O_3_ film. In addition, La-O-W bonding with a peak energy of 532.2 eV was found. For the case of WO_*x*_ phase enhancement, it should not affect the EOT as it can be considered as part of the metal electrode; on the other hand, the effects of La-O-W bonding have never been explored, and it should have some impact in making the effective EOT thicker.

### Silicon/high-*k* interface

**Figure 3:**
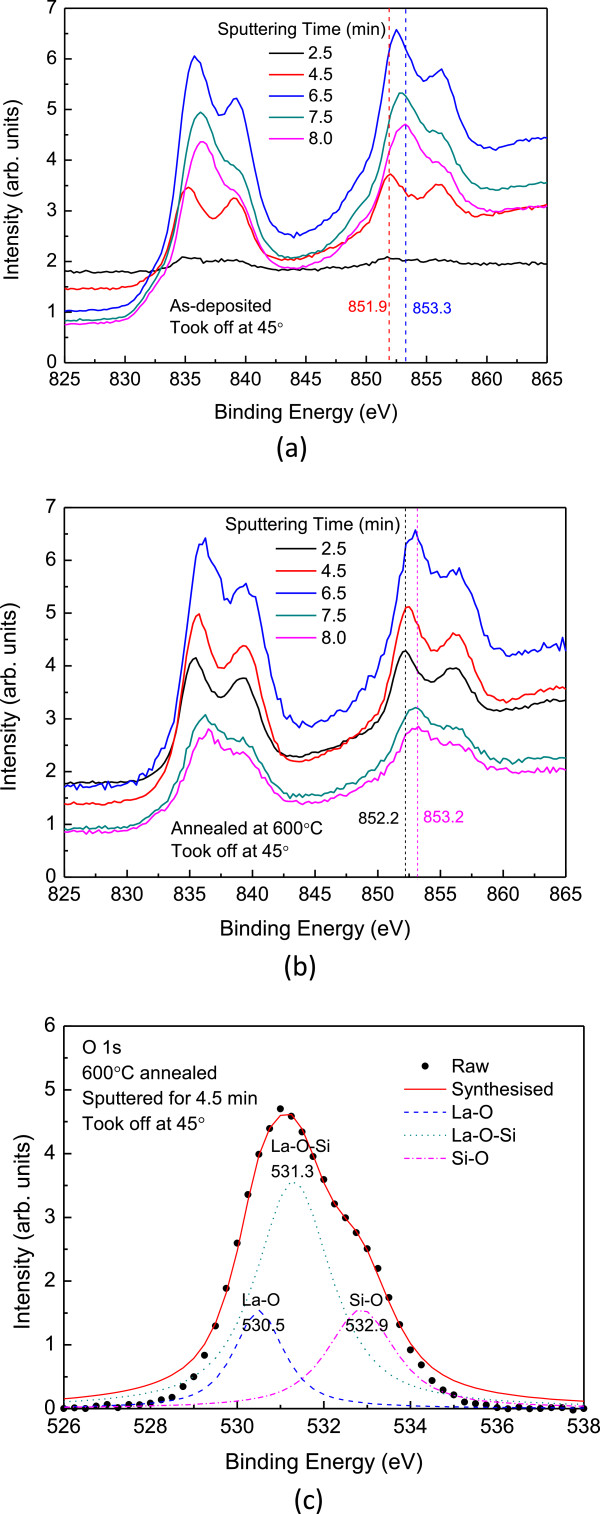
**XPS results showing the existence of interfacial silicate layer at the La**_**2**_**O**_**3**_**/Si interface. (a)** La 3d spectra of the as-deposited sample. **(b)** La 3d spectra of the thermally annealed sample. **(c)** O 1s spectrum taken from the La_2_O_3_/Si interface region for the annealed sample.

**Figure 4:**
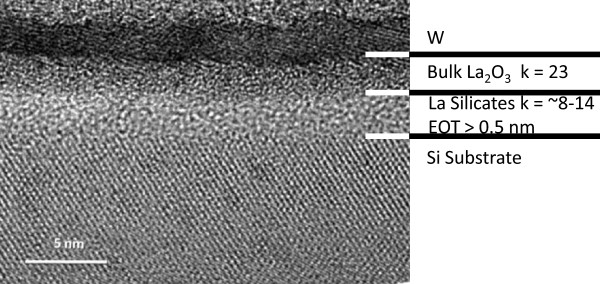
**A TEM picture showing the cross-sectional view of the W/La**_**2**_**O**_**3**_**/Si stack.** A silicate layer of about 2 nm thick was found.

High-*k* can react, especially in the presence of a silicon oxide layer, with the silicon substrate, and the electronic bonding structure at the La_2_O_3_/Si interface should be much more complicated than the SiO_2_/Si case. It was known that the interface bonding may lead to either an insulating layer (silicate bonding) or conductive layer (silicide bonding) [1,2]. Most of the high-*k* silicides are conductive. The interfacial silicide layer will not affect the EOT but the interface metal-Si bonding in the interface trap precursors and results in the channel mobility degradation and other instabilities [1,15,16]. Most of the high-*k* materials including hafnium oxide and lanthanum oxide are only marginally stable against the formation of silicates. The device properties can be improved with the interfacial silicate layer [1]. However, this layer has much smaller *k* values and becomes the lower bound of the thinnest EOT, and needs to be minimized for the subnanometer EOT dielectric. Figure 3 shows the La 3d XPS spectra at different depths. The different depths were obtained by argon sputtering for 2.5 to 8 min, and all the XPS analyses were made at a take-off angle of 45°. This treatment should be able to minimize the artifacts due to ion knock-on effects. The bulk La 3d_3/2_ XPS spectra shows a main peak energy of 851.9 eV and a satellite peak energy of 855.6 eV [1]. As sputtered closer to the substrate, the main peak of La 3d_3/2_ shifts to an even higher energy side of 852.6 eV, and the intensity of the satellite peak becomes weaker, indicating that more silicate with La-O-Si bonding formed at the interface [13]. This trend is more obvious for the sample with thermal annealing (see Figure 3b). Figure 3c depicts the O 1s bonding states near the La_2_O_3_/Si interface for the 600°C annealed sample. With Gaussian decomposition, three oxygen bonding states, i.e., La-O, La-O-Si, and Si-O, were found. It indicates that the thermal annealing does not only lead to the formation of the interfacial silicate layer, but also results in the Si substrate oxidation. Figure 4 depicts the cross-sectional view of the W/La_2_O_3_/Si structure for the sample annealed at 600°C for 30 min; a thick silicate layer of about 2 nm was found at the interface. This thickness of layer is quite substantial as the original film thickness is 5 nm only. With capacitance-voltage measurements, the *k* value of this layer is estimated to be in the range of 8 to 13. Thus, from the EOT point of view, this layer contributes over 0.5 nm of the total thickness. In addition, the interface roughness was significantly increased which led to further channel mobility degradation. Hence, although some of the device properties may be improved by forming the interfacial silicate layer and SiO_2_ layer, the silicate or SiO_2_ layer has much smaller *k* value and becomes the lower bound of the thinnest EOT. It needs to be minimized for the subnanometer EOT dielectric.

It is further noted that the TEM picture also shows a rough interface between La_2_O_3_ and W. The rough interface should be due to the oxidation of tungsten and the reaction between La_2_O_3_ and tungsten at the interface. Although in real device applications, the W/La_2_O_3_ will not undergo such high-temperature annealing, the interface reaction should still exist in a certain extent as a similar phenomenon was also found in the sample which had undergone post-metallization annealing only [14].

### Thermal budget and process sequences

**Figure 5:**
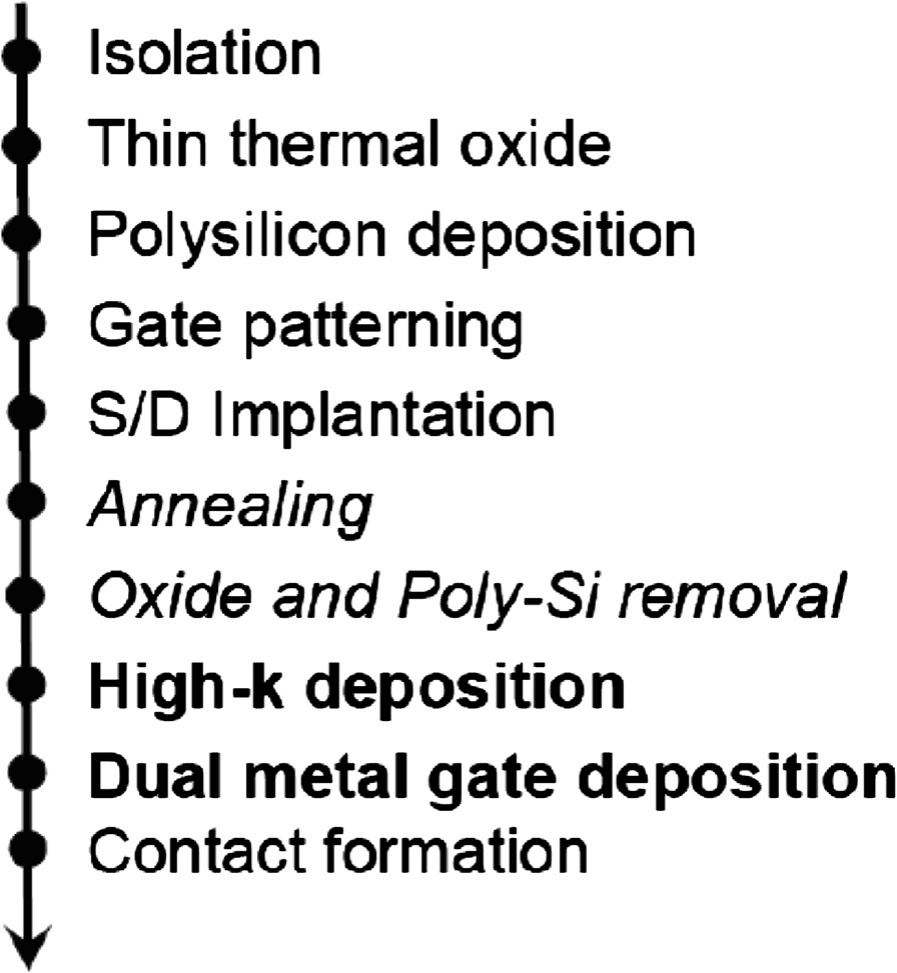
**‘Source/drain first’ process sequence.** This process sequence is for avoiding high-temperature cycles on the as-deposited high-*k* film so as to suppress the growth of the interface silicate layer.

As mentioned, the interface between the high-*k*/Si and thermal stability have become the most challenging issues for next-generation subnanometer EOT gate dielectrics. Unlike silicon oxide or silicon nitride, high-*k* metal oxides are less thermally stable and are easier to be crystallized [1,18]. A low-temperature treatment such as post-metallization annealing (PMA) of about 350°C may still lead to local crystallization of the dielectric [1,18]. Thermal processing above 500°C will result in the interface oxidation and the formation of a interfacial silicate layer. At even higher temperatures, serious crystallization, partial decomposition of metal-oxygen bonds, and phase separation of silicate will occur [1,2]. Thus, the process sequence of a high-*k*-based process has to be adjusted so as to avoid the as-deposited high-*k* material from being exposed at a high-temperature ambient. In addition, to avoid the knock-on of metal atoms into the substrate, the high-*k* film should not be deposited before the ion implantation unless a very thick protection layer is introduced. Several processes, namely, gate-first, gate-last, source/drain first, and combined methods, were proposed [1]. The gate-first process is similar to the conventional one. It requires both the high-*k* and the gate electrode material to be stable at the annealing temperature. In addition, the source/drain doping may produce damages to the gate dielectric also. High-temperature post-implant annealing will also result in the growth of the interfacial layer at the high-*k*/Si interface. The high-temperature process also led to the non-uniformity of the film thickness. Hence, the gate-first process cannot be used with the subnanometer EOT gate dielectric in the deca-nanometer CMOS technology. In the gate-last process, the high-*k* dielectric was deposited and then an intermediate poly-Si layer was deposited and patterned. After the source/drain implantation and salicidation process, the poly-Si gate was replaced with the metal gate. This process avoids the possible knock-on of the high-*k* metal into the substrate and minimizes the number of high-temperature cycles on the gate material. However, this process still causes the high-*k* layer to be exposed to high temperatures. This drawback was resolved with the ‘source/drain first’ process [19]. Figure 5 shows a modified source/drain first process sequence for high-*k* integration. This process reduces the interfacial low-*k* layer growth and seems to be a viable option for preparing the ultimate EOT dielectric film although there are some disadvantages associated with this process sequence re-shuttling.

## Conclusions

In future technology nodes, the gate dielectric thickness of the CMOS devices will be scaled down to the subnanometer range. Lanthanum-based dielectric films have been considered to be suitable candidates for this application. This work presented a detailed study on the interface bonding structures of the W/La_2_O_3_/Si stack. We found that thermal annealing can lead to W oxidation and formation of a complex oxide layer at the W/La_2_O_3_ interface. For the La_2_O_3_/Si interface, thermal annealing leads to a thick low-*k* silicate layer. These interface layers will become the critical constraint for the smallest achievable EOT, and they would also cause a number of instability issues and induce device performance degradation. These issues can be minimized by lowering the thermal budgets and re-shuttling the process sequences.

## Competing interests

The authors declare that they have no competing interests.

## Authors’ contributions

HW generated the research idea, analyzed the data, and wrote the paper. JZ and HJ were involved in some of the sample preparation and TEM experiments. JeZ performed the XPS analysis. KK and HI provided the samples. HW has given final approval of the version to be published. All authors read and approved the final manuscript.
